# Recent Strategies to Combat Biofilms Using Antimicrobial Agents and Therapeutic Approaches

**DOI:** 10.3390/pathogens11030292

**Published:** 2022-02-25

**Authors:** Looniva Shrestha, Hai-Ming Fan, Hui-Ren Tao, Jian-Dong Huang

**Affiliations:** 1 Institute of Synthetic Biology, Shenzhen Institute of Advanced Technology, Chinese Academy of Sciences, Shenzhen 518055, China; jdhuang@hku.hk; 2 Department of Spinal Surgery, Shenzhen University General Hospital, Shenzhen 518055, China; haiming528@szu.edu.cn (H.-M.F.); huiren_tao@szu.edu.cn (H.-R.T.); 3 School of Biomedical Sciences, Li Ka Shing Faculty of Medicine, University of Hongkong, Hong Kong, China

**Keywords:** biofilm, exopolymeric substance, quorum sensing, antibiofilm agents, antibiotic tolerance

## Abstract

Biofilms are intricate bacterial assemblages that attach to diverse surfaces using an extracellular polymeric substance that protects them from the host immune system and conventional antibiotics. Biofilms cause chronic infections that result in millions of deaths around the world every year. Since the antibiotic tolerance mechanism in biofilm is different than that of the planktonic cells due to its multicellular structure, the currently available antibiotics are inadequate to treat biofilm-associated infections which have led to an immense need to find newer treatment options. Over the years, various novel antibiofilm compounds able to fight biofilms have been discovered. In this review, we have focused on the recent and intensively researched therapeutic techniques and antibiofilm agents used for biofilm treatment and grouped them according to their type and mode of action. We also discuss some therapeutic approaches that have the potential for future advancement.

## 1. Biofilms and Chronic Infections

**Figure 1:**
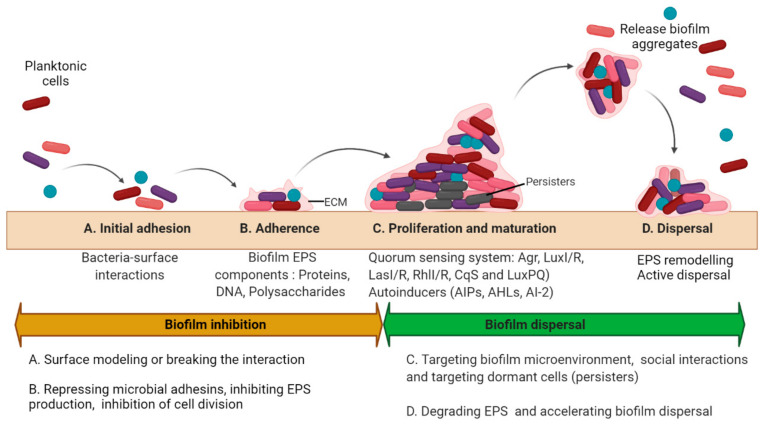
Biofilm development and antibiofilm strategies. The microbial biofilm cycle could be classified into 4 phases: Initial attachment, Adhesion, Maturation, and Dispersal. The biofilm inhibitory and dispersal strategies are summarized as per the stages in biofilm development. (**A**) The initial attachment can be disrupted by interfering with the interactions between the surface and the microorganism either by surface remodeling or physical removal of the biofilms; (**B**) Adhesion can be inhibited by targeting biofilm EPS and cellular division; (**C**) Disruption of biofilms in proliferating and maturing phase may be accomplished either by physical removal or by damaging the EPS matrix primarily by affecting the formation of pathogenic microenvironments (such as hypoxia or low pH), and quorum sensing along with the eradication of persister cells. (**D**) Biofilm dispersal could be achieved by remodeling the EPS matrix or accelerating the dispersal mechanisms. (Different colors of the cells represent different bacteria within the biofilm. Circular cells represent cocci and rod shaped cells represent bacilli).

Biofilms are multicellular clusters of microbes that adhere to various surfaces using an extracellular polymeric substance (EPS) made up of proteins, polysaccharides, or extracellular DNA (eDNA) [1]. The EPS provides mechanical strength, shelter from antimicrobials and host immune cells, attachment and clumping of biofilm cells, tolerance to dehydration, and assimilation of different compounds, and also provides a carbon source at nutrient-deprived conditions [2]. The capacity to form biofilms is a general feature of bacteria [3]. All eukaryotes are colonized by microorganisms that form biofilms. The biofilm microorganisms elicit definite mechanisms for early adherence to a surface, growth, and expansion of a community structure and microenvironment, and dispersal. The molecular mechanism controlling biofilm formation differs greatly amid different species and even fluctuates between separate strains of the identical species [3]. However, there are some common mechanisms for biofilm formation (see Figure 1). The microbial biofilm cycle consists of four phases: A. Initial attachment, where the planktonic cells’ initial attachment to the medical device surfaces or the host is through bacteria−surface interactions that are ultimately determined by the interplay between physicochemical interactions. B. Adherence is a stage where microbes adhere to a medical device or the host through adhesins. In this phase, microbes start to divide and form an exopolymeric substance (EPS), which improves adhesion while the formation of an EPS envelops the cells. C. Proliferation and maturation phase are whereby 3D biofilm assemblies develop where the EPS offers multifunctional and concealing microenvironments where diverse microorganisms can coexist and communicate through a quorum sensing (QS) system. D. Dispersal is the final stage where the cells escape from the biofilm to re-enter the planktonic phase [4]. 

Biofilm infections include both device-related and non-device-related biofilms that affect numerous people in the world each year that result in numerous deaths [5]. The microorganisms that are most frequently associated with medical devices are the staphylococci (particularly *Staphylococcus epidermidis* and *Staphylococcus aureus*), followed by *Pseudomonas aeruginosa* [6]. The microbes can also gain access to the host body through the contamination of therapeutic devices such as catheters, contact lenses, prosthetic heart valves and joints, voice prostheses, and pacemakers [7,8]. Biofilm production on indwelling therapeutic devices significantly affects surgical and instrumental procedures and public health as well. Wound infections, cystic fibrosis, otitis media, native valve endocarditis, bladder infections, and periodontitis are examples of non-device-related infections. The mucous layer in the host segregates bacteria from direct contact with the epithelium. Nonetheless, any rupture in the mucous layer exposes bacteria to the host epithelium and infection of mucosal surfaces [6]. To survive inside the host, the invading microbes need to overcome the epithelial wall, host-microbiome, a variety of leukocytes, and complement [2]. The immune system identifies diverse bacterial molecular patterns, though these elements could be concealed in the biofilms [2]. Biofilms decrease the efficiency of both macrophages and polymorphonuclear neutrophils [2]. This results in chronic mucosal diseases such as inflammatory bowel diseases, pharyngo-tonsillitis, rhinosinusitis, urethritis, and vaginitis [6]. The current therapeutic approaches used by medical staff include aggressive physical removal of biofilms and localized delivery of high and sustained antimicrobial chemotherapy such as antibiotics. Intravenous catheters are usually treated using a “lock therapy” which involves the treatment of a high dose of antibiotics into the lumen of the catheter for several hours [4]. Biofilms-related problems are increasing in the health care, food industry, and other fields while new antibiotics have not been produced by the pharmaceutical industry in more than a decade. Furthermore, most biofilm bacteria are tolerant to antibiotics [9]. Thus, there is an urgent need to find an alternative to antibiotics for treating biofilm-related infections. 

## 2. Biofilm Antibiotic Tolerance

**Figure 2:**
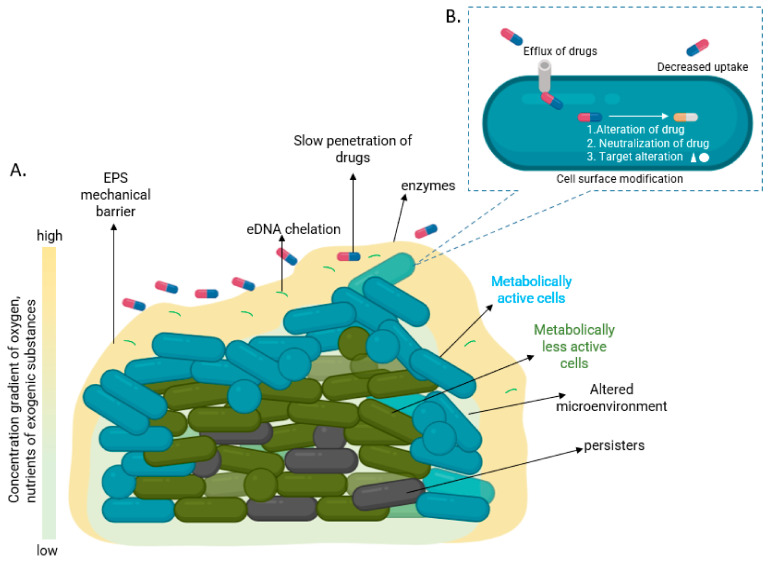
Diagrammatic representation of antibiotic (drug) tolerance in biofilms. Possible tolerance mechanisms at (**A**) community and (**B**) cellular level.

Antibiotic resistance is the acquired resistance to antibiotics through inheritable genetic mutations. In contrast, antibiotic tolerance is a physiological state of biofilm cell populations that is temporary and non-heritable [10]. Bacterial biofilms are tough to cure because of their antibiotic tolerance and might pave the way to chronic infections [11,12]. Antibiotic tolerance mechanisms in biofilm might be different from the planktonic cells [12]. The biofilm entities have a few extra tolerance mechanisms than the planktonic counterparts which hinder the treatment options and lead to the development as well as distribution of persistent bacteria [12] (Figure 2). The common causes of antibiotic failure in biofilms include: 1. Inhibition of antibiotic penetration caused by EPS barrier; 2. Accumulation of antibiotic degrading enzymes in the EPS; 3. Presence of eDNA (Tolerance to antibiofilm agents caused due to changes in the outer membrane induced by eDNA via chelating cations and resistance to antibiotics by eDNA mediated horizontal gene transfer of antibiotic resistance genes between biofilm microorganisms); 4. Quorum sensing within the biofilms that control biofilm volume, thickness, roughness, and channel formation; 5. Upregulation of efflux pumps and inactivation of drug by a. alteration of either drug or target and b. neutralization of drug; 6. Multispecies interaction and microbial diversity; and 7. Altered growth rate, stress response, and persister cells [10]. Deep inside the biofilm, dormant microorganisms known as persisters are present which are linked to a common stress response. The reduced metabolism protects the bacteria from the impacts of pH variations, osmolality, and chemical concentration [13]. Persisters are of immense clinical concern, as they play a crucial part in the antibiotic failure, relapse of bacterial infections, and serve as a pool from which resistant strains could emerge [14]. Additionally, biofilms include many concentration gradients, creating aerobic and anaerobic microenvironments which increase tolerance to antiseptics and antibiotics treatment [13]. Therefore, biofilm antibiotic tolerance is due to its multicellular nature and any interruption in step in multicellular structure might increase the antibiotic effectivity and host defenses [9].

## 3. Basic Strategies to Treat Biofilms

In general, two different strategies to treat biofilms exist: (1) biofilm inhibition, to prevent biofilm from forming, and (2) biofilm dispersal, to eliminate already formed biofilms (Figure 1). In order to avoid biofilm development, the adherence of planktonic cells to various surfaces or the development into premature microcolonies and also to matured biofilms should be prevented. The early attachment could be tackled in two ways: Firstly, one can modify the surface that imprints distinct 3D patterns or target physicochemical properties such as surface hydrophobicity to block microbial attachment [15,16,17]. Secondly, one can pre-condition the surfaces with chemicals to prevent initial attachment. For example, treating the surface with certain surfactants can inhibit bacterial adherence [18]. The approaches that are aimed to impede bacterial establishment on various surfaces or to avoid maturation of biofilms, typically exploit some stimuli to control the genes involved in the formation of biofilm [19] such as repressing the expressions of bacterial adhesins [20], inhibition of the biofilm EPS synthesis [21], and provoking Quorum sensing (QS) signals [22,23,24,25], or even eliminate bacteria in biofilms [26].

**Figure 3:**
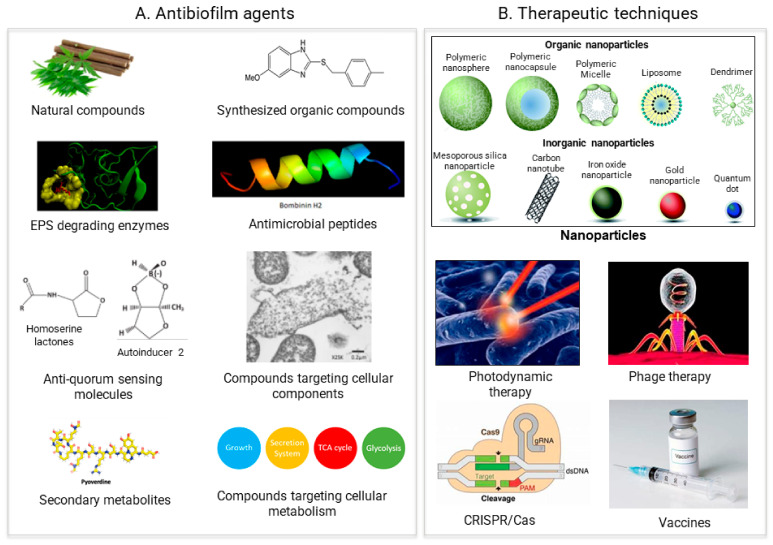
Recent approaches for biofilm treatment. Biofilms could be controlled by using (**A**) Antibiofilm agents that target various compounds involved in biofilm formation (**B**) Therapeutic methods directly targeting the biofilm formation process.

Preformed matured biofilms could be dispersed by breaking off biofilm assemblies and promoting detachments (Figure 1). For inducing biofilm detachment, EPS polymers should be disrupted, or cells should be programmed to disperse [17,27]. All of the antibiofilm agents and the therapeutic approaches we discuss to tackle biofilms are based on these two strategies (Figure 3).

## 4. Antimicrobial Agents

Antibiofilm agents belong to diverse compounds that can inhibit and eradicate biofilm formation. The established anti-biofilm compounds are chiefly extracted from natural sources while various chelating agents and synthetic compounds have been found to exhibit some anti-biofilm activity. A current review by Plakunov et al. recommended the agents to be classified into four categories based on their actions in various stages of biofilm formation (Table 1) [28]. This rule of categorization seems suitable to subcategorize a certain class of compounds but rather complicated to classify a wide range of antimicrobial compounds en bloc. In this review, we have categorized each agent based on their mode of action and their type. Here, we review some contemporary antibiofilm agents and the therapeutic approaches that can be used as alternatives to antibiotics for treating biofilms.

### 4.1. Surface Attachment Inhibitors 

The initial phases of attachment are very crucial in the biofilm development process. The control of surface attachment can inhibit the whole process of biofilm development. Biofilm formation can also be inhibited by the inhibition of adhesin and EPS molecules. When the bacteria have short-range interaction with the surface, the hydrophobic interactions, dipole, ionic, and hydrogen bonds begin to dominate over some other interactions, and then bacteria start to attach through the cellular or molecular phase [29] (Figure 1). Surfactants are the popular choice of antimicrobial agents for inhibiting bacterial adhesion to the surfaces as they decrease the interfacial tension between two substances. Surfactants are amphiphilic as they comprise of hydrophilic and hydrophobic moiety and at the same time, they can be categorized as non-ionic, anionic, cationic, and amphoteric surfactants [27]. Triton X-100 and Tween 80 (Polysorbate 80) are the two popular non-ionic, synthetically derived, and regularly used surfactants in laboratories (Table 2). Tween 80 decreased *S. aureus* medical device associated biofilm development at concentrations safe in humans [30]. Triton X-100 could stimulate autolysis by enhancing bacterial vulnerability to antibiotics and altering the architecture and physiological features of biofilms by reducing the protein and carbohydrate constitution in the EPS [31,32]. Biosurfactants are the surface-active compounds created by microorganisms that comprise structurally diverse biomolecules [33]. Cationic surfactants such as quaternary ammonium compounds (QACs) are used as disinfectants within the food industry and used in several medical conditions. QACs bind to negatively charged areas in microbes that cause stress to the cell wall, lysis, and cell death. QACs could also cause protein denaturation that affects cell wall permeability and reduces the uptake of nutrients. Non-ionic-based surfactants incorporating poloxamer 188, are regarded as non-cytotoxic and therefore represent a useful combination in wound care. Numerous studies using antimicrobials together with poloxamers showed enhanced antimicrobial efficacy [34]. Similar to regular surfactants, numerous biosurfactants have antimicrobial activities while some even seem to prevent surface colonization by pathogens [27]. One of them is rhamnolipid, which is the chief glycolipid formed by several bacterial species, chiefly by *P. aeruginosa* [35], and stimulates biofilm dispersal in *P. aeruginosa*, *S. aureus*, *Salmonella enteritidis*, and *Listeria monocytogenes* [36]. Furthermore, rhamnolipids from *P. aeruginosa* W10 were also known to disperse biofilms of various industrial bacterial strains on the pipelines [37]. Since biosurfactants are usually associated together with isomers and cogeners and rarely in pure form, the purification process could be exhaustive and expensive [38]. At the same time, they could be very cytotoxic and hemolytic due to their activity with cellular membrane [39]. Therefore, their use for controlling biofilms could be limited to coating medical devices and anti-adhesive agents.

### 4.2. Compound Inducing Cell Lysis 

To inhibit the biofilm formation process, it is best to kill the bacteria in the earlier phases of biofilm development. It can be achieved through targeting the cellular components and mechanisms. The breakdown of peptidoglycan that makes the cell wall of bacteria could inhibit biofilm formation as it changes the constitution of teichoic acids and proteins on the cell wall and likewise releases the signals that regulate genes related to biofilm [54]. Enzymes such as transglycosylase and peptidoglycan hydrolases (endolysins) break the cell wall and often result in bacterial cell death [55,56].

Cell division is critical for bacterial existence in the biofilms and for spreading further to new surfaces [54]. Chelating agents such as Ethylenediaminetetraacetic acid (EDTA) can damage the cell wall, subsequently disrupting the biofilms via sequestering zinc, magnesium, iron, and calcium [40]. EDTA is generally safe for use in prescription medicine and in small amounts in food preservatives. Similarly, Chitosan is a natural polymer used in numerous applications in the biomedical field because of its biodegradability, bioadhesive property, and bioactivity [56]. It is known to disrupt negatively charged cell membranes due to its cationic nature (Table 2) [41]. Therefore, by using such agents we can tackle the bacteria in the early phase of biofilm development.

### 4.3. Antiquorum Sensing Molecules

QS is a cell–cell interaction mechanism in the microbial groups to harvest a coordinated effort on regulating the genes related to virulence, biofilm formation, antibiotic tolerance, and survival. The QS mechanism is brought by a set of two proteins in Gram-negative bacteria. One of these is an autoinducer (AI) which leads to signaling molecule production, while the other one acts in response to the AI [57]. In contrast to Gram-positive bacteria that use secreted autoinducing peptides (AIPs) such as AIs for QS, Gram-negative bacteria employ homoserine lactones (HSL). In addition, unlike Gram-negative bacteria that use a regulator-type protein as an autoinducer sensor, Gram-positive bacteria employ two-component adaptive response proteins for sensing AIs. This mechanism of signaling is stimulated by a phosphorylation/dephosphorylation cascade [58]. Cyclic di-GMP (c-di-GMP) is also known as a secondary messenger since it is responsible for controlling several functions such as motility, cell cycle, differentiation, developmental transitions, adhesion, aggregation, biofilm production, and virulence in various pathogens. In several Gram-negative bacteria such as *Escherichia coli*, *Gluconacetobacter xylinus*, *P. aeruginosa*, and *Salmonella enterica*, c-di-GMP plays a significant role in the shift in between motile to sessile lifestyle, construction of three-dimensional (3D) biofilms, and during biofilm dispersal [59,60,61]. 

Many natural and synthetic compounds act as anti-QS molecules that target various QS signaling molecules (Table 3). A natural compound such as garlic was able to reduce the virulence factors progression and decrease the QS signal production in *P. aeruginosa* in a mouse urinary tract infection (UTI) model [62]. In another study by Persson et al., it was reported that garlic extracts inhibit biofilm production in six clinical isolates of bacteria [57]. Besides, through the rigorous design and screening, all the biological compounds from a potent QS inhibitor interrupted QS signaling by negatively regulating the transcriptional regulators LasR and LuxR [63]. Ichangin and isolimonic acid are the strong regulators of cell–cell signaling in bacteria, while they are the effective repressors of biofilm and the type III secretion system. Moreover, isolimonic acid also seems to affect AI-3/epinephrine generated cell–cell signaling pathways in QseA and QseBC dependent manner [64,65]. It also interfered in AI-2 based QS by reducing the LuxR DNA-binding potential in several *Vibrio* spp. [65]. Another natural compound cinnamaldehyde was known to reduce *E. coli* swimming motility and alter biofilm structure and formation [66]. It was also found that hordenine, a potent phenylethylamine alkaloid obtained from barley, exhibits a dose-dependent decline in the production of the signaling molecule and affects biofilm production in *P. aeruginosa* [67]. Furthermore, hordenine also effectively reduces QS-associated gene expression and virulence factors of *P. aeruginosa* PAO1 [67,68]. This suggested that hordenine appears to be a novel anti-QS agent that could protect from pathogens [67]. Plant polyphenols known as quercetin are reported to significantly reduce biofilm production and other virulence factors at a lower concentration than formerly known substances and plant extracts [69,70,71,72]. Furthermore, a study on QS-associated transcriptional changes revealed that LasI/R, RhlI/R expression levels involved in QS were significantly decreased [73]. Autoinducing peptide type I (AIP-I) stimulated MRSA biofilms dispersal on titanium disks, causing detached MRSA more vulnerable to treatment with rifampin and levofloxacin [74]. RNAIII-inhibiting peptide (RIP) resulted in a 7-log reduction in MRSA in a mouse wound model [75]. While the increased effectiveness of antibiotic treatment with QSI in vivo is promising, reduced bacterial loads often depend on the strain and biofilm model [4].

Naturally, nitric oxide (NO) is recognized as the universal signaling molecule that can circulate easily in biological systems. However, some studies highlight the role of NO in biofilm dispersal by targeting the QS system of bacteria [27]. The NO producing agents including sodium nitroprusside (SNP) induced lifestyle transition in bacteria, from the sessile biofilm state to a mobile planktonic state by reducing the amount of intracellular c-di-GMP, thereby causing dispersal of *P. aeruginosa* biofilms [76]. A similar effect of biofilm-dispersal by NO donors has been verified in *Bacillus subtilis* as well [77]. These studies underscore that NO generating agents could be potential antibiofilm agents.

### 4.4. Synthetic Small Organic Molecules 

The design of synthetic small organic molecules has paved a new route to overcome antibiotic tolerance and interfere with biofilms [78]. It has drawn remarkable attention in the past few decades. Numerous research shows that small organic molecules inhibit biofilms by different modes of action (Table 4). 

Some imidazole and benzimidazole compounds are able not only to inhibit biofilms but also to disperse them. The molecular mechanism behind the antibiofilm effect for the 5-phenyl-2-aminoimidazole was interpreted in *Salmonella typhimurium* [79]. The study emphasizes the potential of 5-phenyl-2-aminoimidazole to decrease the expression of CsgD, and adrA and csgB genes regulated by it, thus preventing the biofilm EPS formation [79]. Sambanthamoorthy et al. synthesized a 5-methoxy-2-[(4-methyl-benzyl) sulfanyl]-1H-benzimidazole, commonly known as ABC-1 (antibiofilm compound-1) which showed an antibiofilm effect against the Gram-negative bacteria *Vibrio cholera* and *P. aeruginosa* [80]. ABC-1 was also able to inhibit biofilms in Gram-positive pathogens including *S. aureus* at lower concentrations by targeting eDNA, polysaccharide intercellular adhesion (PIA), and Protein A (SpA) expression [1]. Likewise, Frei et al. confirmed the strong effect of the 5, 6- dimethoxy-2-aminobenzimidazole not only inhibited *P. aeruginosa* biofilms but also dispersed them by targeting two QS receptors, LasR and RhlR [81]. 

Pyrazole is an exceptional aromatic heterocyclic compound with five-membered rings, is also known to be a biofilm modulator. Suresh et al. tested three Pyrazolo-pyrimido [4,5-d] pyrimidines (compounds 19 a–c) and found that it was able to inhibit Gram-positive bacteria including *S. aureus*, *B. subtitlis*, and *Microococcus luteus* [82]. Remarkably, biofilm treatment with compound 19b displayed a substantial surge in intracellular ROS levels in *M. luteus* at the dose of 0.5 μg/mL, which caused the cells to undergo oxidative stress that caused membrane damage leading to cell lysis and death [83,84].

Indole derivatives are known to repress motility, chemotaxis, and adhesion in *E. coli*. In a screening of six plant and animal derivatives of indole, indole-3-carboxaldehyde and 3-indolylacetonitrile were found to be potential biofilm inhibitors against *P. aeruginosa* and *E. coli* O157: H7. These compounds decrease biofilms by reducing curli production without affecting microbial growth [85,86].

2-Phenylhydrazineylidene derivatives can prevent bacterial adhesion by Sortase A (SrtA) inhibition [87], a transpeptidase enzyme that aids in biofilm production by incorporating cell-surface proteins into the Gram-positive bacteria cell wall. Inhibition of SrtA is also associated with loss of virulence factors in *S. aureus*, including attenuation in the binding potential to fibrinogen, and fibronectin, lgG, along with a decrease in biofilm formation [1,88,89]. Pyrrole derivatives are also found to inhibit biofilms in Gram-positive pathogens. For example, Dihydro-pyrrol2-ones (DPO) derivatives such as diethyl1-(3-chlorophenyl)-4-((3-chlorophenyl) amino)-5-oxo-2,5-dihydro-1H-pyrrole-2,3-dicarboxylate exhibited inhibition in *P. aeruginosa* growth and biofilm formation by inhibiting mannitol dehydrogenase (MDH) and eDNA. MDH is involved in the synthesis of alginate which is one of the EPS components of *P. aeruginosa* [90].

Brominated furanone derivatives are known to inhibit biofilms in different bacterial species. The synthetic (Z)-5-bromomethylene-2(5H)-furanone repressed microbial communication mediated by AI-2 in several Streptococci such as *Streptococcus angionus*, *Streptococci intermedius*, and *Streptococcus mutans*. Similarly, bicyclic brominated furanones inhibited AI-2 mediated QS in *Tannerella forsythia*, *Porphyoromonas gingivalis*, and *Fusobacterium nucleatum* [91]. 

Halogenated phenazines showed powerful activity against Methicillin-resistant *S. epidermidis* (MRSE), Methicillin-resistant *S. aureus* (MRSA), and Vancomycin-resistant *Enterococci* (VRE) by binding with iron (II) and copper (II) that exhibited antibiofilm activity [92]. In the past, several bacterial infections were treated with quinolones. However, using an in silico virtual screening method, it has been lately identified that quinolone compound Ia could reduce *P. aeruginosa* biofilms by inhibiting PqsR (regulatory proteins). It also showed a synergistic effect with other antibiotics such as tobramycin [93]. Sommer et al. showed the 3,4-dimethoxycinnamide derivative showed biofilm inhibition in *P. aeruginosa* by inhibiting LecB [94]. 

Although several small molecules have proved to be efficient biofilm inhibitors, none of these agents have reached clinical use due to a lack of experiments in animal models. Therefore, new in vivo studies using small molecules are urgently needed to assess their therapeutic potential [78].

**Table 4:** Small organic molecules with the known biofilm inhibition or eradication mechanism.

	Small Organic Molecules	Biological Role	Effective Against	Type of Compound	Reference
1	5-phenyl-2-aminoimidazole	Reduction in transription of CsgD, csgB and adrA	*S. typhimurium*	Imidazole derivative	[79]
2	ABC-1	Reduction in SpA and PIA production and decrease eDNA release	*S. aureus*, Gram-positive and Gram-negative pathogens	Imidazole derivative	[1,80]
3	5,6-dimethoxy-2-aminobenzimidazole	Reduction in QS receptors (LasR and RhlR)	*P. aeruginosa*	Imidazole derivative	[81]
4	Pyrazolo-pyrimido [4,5-d] pyrimidines	ROS accumulation, loss of membrane integrity	*M. luteus*, *S. aureus*, *B. subtilis*, *E. coli*, *K. planticola*	pyrazole compound	[82,83,84]
5	Indole-3-carboxaldehyde and 3-indolylacetonitrile	Reduction of curli production	*E. coli*, *P. aeruginosa*	indole and carbazole derivative	[85,86]
6	Phenylhydrazine analogues	SrtA inhibition	*S. aureus*	2-Phenylhydrazineylidene derivatives	[87]
7	Diethyl 1-(2-chlorophenyl)-4-((3-chlorophenyl)amino)-5-oxo-2,5-dihydr-1H-pyrrole-2,3-dicarboxylate	MDH and eDNA inhibition	*P. aeruginosa*	Pyrrole derivatives	[90]
8	(Z)-5-bromomethylene-2(5H)-furanone	AI-2-mediated QS inhibition	*S. anginosus*, *S.intermedius* and *S. mutans*	Furanone and oxazolidinone derivatives	[91]
9	Bicyclic brominated furanones	AI-2-mediated QS inhibition	*F. nucleatum*, *P. gingivalis* and *T. forsythia*	Furanone and oxazolidinone derivatives	[91]
10	Halogenated phenazine	Bind with copper (II ) and iron (II)	MRSA, MRSE, and VRE	Phenazine and quinolone derivative	[92]
11	Quinolone compound Ia	PqsR inhibition	*P. aeruginosa*	Quinolone derivative	[93]
12	3,4-dimethoxycinnamide derivative	LecB inhibition	*P. aeruginosa*	Cinnamide derivative of d-mannose	[94]

### 4.5. Secondary Metabolites 

Secondary metabolites (SM) do not directly contribute to the basal metabolism of its producing organism instead act as essential factors to either attract, repel, or kill other organisms and thereby increase the chance of self-survival [42,95]. Unique secondary plant metabolites such as *Citrus limonoids* presented their potential to affect biofilm formation and cell–cell signaling in *Vibrio harveyi* by modulating the expression LuxO, but not the promoter activity of LuxR (Table 2) [42]. 

Since marine organisms are a rich source of novel bioactive metabolites, studies on marine fungal and bacterial secondary metabolites have been gradually growing for the development of novel therapeutic agents [96]. For example, a secondary metabolite identified as cyclo(l-Tyr-l-Leu) produced from a marine ascomycete *Penicillium* spp isolated from the sponge *Axinella corrugata* which inhibited biofilm formation by *S. epidermidis* [43]. Similarly, Park et al. described the discovery of three novel secondary peptidic metabolites known as cahuitamycins from *Streptomyces gandocaensis* that were the inhibitors of *Acinetobacter baumannii* biofilms [44]. Thus, secondary metabolites could be abundantly found where the microorganisms coexist together. This highlights that nature could be an unlimited source for drug discovery.

Seaweed secondary metabolites such as phlorotannin possess antibacterial properties. Several in vivo studies and clinical trials exist on the health benefits of Phlorotanin. However, these studies were not based on the antibiofilm properties of phlorotannin [45,97]. The current studies in vitro show antibiofilm properties of secondary metabolites, but more in vivo studies on the antibiofilm properties seem to be required. 

### 4.6. Antibiofilm Peptides

Antimicrobial peptides (AMPs) are a type of innate defense mechanism in different eukaryotes which was first discovered by Kiss and Michl in the 1960s. AMPs are cationic and hydrophobic residues containing molecules that can interact with various microorganisms such as bacteria, fungus, protozoa, and some enveloped viruses [98,99,100]. Since AMPs have low antigenicity and rapid killing effect in comparison to conventional antibiotics, it has been showing momentous potential in recent years [101]. Studies show sub-minimal inhibitory concentration (MIC) of some AMPs is also able to inhibit biofilm in various pathogens, thus these peptides are termed antibiofilm peptides (ABPs) [102]. Antimicrobial peptides display a wide range of antibiofilm effects by (1) cleavage of peptidoglycan, (2) change of membrane permeabilization or membrane potential, (3) neutralization or disassembly of lipopolysaccharides, (4) inhibition of cell division and cell survival, (5) modulate the synthesis of adhesion molecule synthesis and function, and (6) repression of the stringent response of the bacteria [54,103]. Some ABPs and their mechanisms for biofilm inhibition or dispersal are given in Table 5. 

AMPs such as nisin and bovicin HC5 reduced the *S. aureus* adhesion to the polystyrene surfaces and altered the cell as well as polystyrene surface hydrophobicity. These AMPs also changed the biofilm-related gene expressions in planktonic cells [104]. On the other hand, a popular human cathelicidin AMP, LL-37, and indolicidin were known to inhibit *P. aeruginosa* biofilm formation. It was likely achieved by inhibiting the transcription of Las and Rhl QS systems [105,106]. LL-37 also inhibits biofilm formation of *P. aeruginosa* by upregulating the expression of genes needed for type IV pili biosynthesis and function [105]. Likewise, peptide 1037 efficiently inhibits biofilm production by the Gram-positive *L. monocytogenes* and Gram-negative pathogens *Burkholderia cenocepacia* and *P. aeruginosa*. Peptide 1037 directly reduced biofilm production by decreasing swarming and swimming motilities, inducing twitching motility, and downregulation of several genes related to biofilm production [106].

Fuente Nunez et al. found an effective anti-biofilm peptide 1018 which could bind and degrade (p)ppGpp, a crucial signal required for the formation of biofilm [102]. Peptide 1018 treatment completely prevented biofilm production at much lower concentrations, which did not alter planktonic growth and also caused degradation of mature biofilms in *E. coli*, *A. baumannii*, *P. aeruginosa*, *Klebsiella pneumoniae*, MRSA, *S. Typhimurium*, and Burkholderia cenocepacia [102]. D-enantiomeric protease-resistant peptides DJK-5 and DJK-6 could reduce (p)ppGpp in biofilms of *P. aeruginosa* to a greater amount than 1018 [107]. 

Apidaecin, pyrrhocoricin, and drosocin are 18–20 amino acid, proline-rich residues that were initially obtained from insects. These AMPs attack a target microbial protein in a stereospecific manner. They interact with the bacterial heat-shock protein DnaK by inhibiting chaperone-assisted protein folding and limiting the DnaK ATPase activity [108,109,110]. The peptide antibiotic microcin B17 (MccB17) is the first peptide known to repress a type II DNA topoisomerase activity. MccB17 blocks *E. coli* DNA gyrase by trapping an enzyme-DNA cleavable complex [111]. PR-39, an AMP which was obtained from the upper portion of a pig’s small intestine, could kill growing bacteria faster than non-growing cells. It is suggested that PR-39 kills bacteria by stopping protein and DNA synthesis [112].

**Table 5:** Antibiofilm peptides with known modes of actions.

Biofilms	AMP	Amino Acid Sequence	MW (g/moL)	No. of Residues	Mode of Action	References
*P. aeruginosa*	LL-37	LLGDFFRKSKEKIGKEFKRIVQRIKDFLRNLVPRTES	4493.33	37	Reduces swimming and swarming motilities, promotes twitching motility, downregulates the genes required for biofilm formation and influences QS system	[105,106]
	1037	KRFRIRVRV	1229.54	9		[106]
	1018	VRLIVAVRIWRR	1536.93	12	binds and degrades (p)ppGpp	[102]
	Esculentin-1a (1–21)	GIFSKLAGKKIKNLLISGLKG	2185.73	21	Disrupts cell membrane	[113]
	RN3(5-17P22-36)	RPFTRAQWFAIQHISPRTIAMRAINNYRWR	3758.38	30	Depolarizes and permeabilize cell membrane	[114]
	S4 (1–16)	ALWKTLLKKVLKAAAK	1782.29	16	disintegrates and release membrane lipids	[115]
	DJK-5	VQWRAIRVRVIR	1551.91	12	degrade (p)ppGpp	[107]
*S. aureus*	Nisin A	MSTKDFNLDLVSVSKKDSGASPR	3354.1	23	Depolarizes cell membrane	[116]
	lacticin Q	MAGFLKVVQLLAKYGSKAVQMAWANKGKILDWLNAGQAIDKVVSKIKQILGIK	5785.05	53		
	Nukacin ISK-1	KK-KSGVIPTVSHGCHMNSFQFVFTCC	2886.44	26		
	HC5	VGXRYASXPGXSWKYVXF	1616.84	14	alter surface hydrophobicity	[104]
*S. epidermidis*	Hepcidin 20	ICIFCCGCCHRSHCGMCCKT	2208.8	20	Acts on Polysaccharide Intercellular Adhesin	[117]
	Human β defensin 3 (HBD-3)	GIINTLQKYYCRVRGGRCAVLSCLPKEEQIGKCSTRGRKCCRRKK	5161.24	45	targets *icaA*, *icaD*, and *icaR* genes	[118]
*S. mutans*	P1	PARKARAATAATAATAATAATAAT	2158	24	interferes and degrades EPS	[119]
*E. coli*	Pyrrhocoricin	VDKGSYLPRPTPPRPIYNRN	2340.67	20	bind with DNaK	[108,109,110]
	Apdidaecin	GNNRPVYIPQPRPPHPRI	2108.44	18		
	Drosocin	GKPRPYSPRPTSHPRPIRV	2198.56	19		
	Microcin B17	VGIGGGGGGGGGGSCGGQGGGCGGCSNGCSGGNGGSGGSGSHI	3255.35	43	Inhibition of DNA replication by inhibiting type II DNA topoisomerase	[111]
	PR-39	RRRPRPPYLPRPRPPPFFPPRLPPRIPPGFPPRFPPRFP	4720.7	39	stop the synthesis of DNA and protein	[112]

Some antimicrobial peptides polarize and permeabilize the cell membrane and finally cause membrane disruptions. Esculentin (Esc (1–21) is the AMP derived from the frog skin. It could breach the cytoplasmic membrane of Gram-negative bacteria for example *P. aeruginosa* PAO1 inside the biofilms and lead to a discharge of β-galactosidase. However, this impact on biofilm cells was slower when compared to the planktonic cells [113]. Similarly, another synthesized peptide, RN3(5-17P22-36) was acquired from the cationic proteins of eosinophil granules. It was also capable of causing cell lysis by membrane disruption in biofilms. There was a 2–3-fold reduction in membrane depolarization in the biofilm cells as compared with the planktonic cells at the same dose of AMP [114]. Another peptide, known as S4 (1–16) M4Ka, which is a derivative of dermaseptin S4 also causes the flux of membrane lipids, bacterial dispersal, thus leading to a reduction in *P. aeruginosa* biofilm [115]. Bacteriocins such as nukacin ISK-1, lacticin Q, and nisin A also destroyed the membrane potential of *S. aureus* biofilm cells and caused the ATP flux from the cells [116].

Several AMPs also target biofilm EPS components. AMPs such as P1 could destroy biofilm EPS formed by *Streptococcus mutans* that resulted in decreased biofilm formation on polystyrene and saliva-coated hydroxyapatite [119]. Another AMP, hepcidin 20, was able to decrease EPS load and affect the biofilm structure of *S. epidermidis* by inhibiting PIA [117]. Similarly, Human β -defensin 3 (HBD-3) induced downregulation of ica operon genes, such as *icaA* and *icaD*, and at the same time upregulated *icaR* genes of *S. epidermidis* thus decreasing PIA production resulting in biofilm inhibition [118]. As all of these results suggest antibiofilm peptides can control biofilms using several different kinds of mechanisms, these peptides can be further classified into several sub-groups as suggested by Plakunov et al. [28].

A few antibiofilm peptides have even been used in treating biofilms in animals and agriculture. Since antibiofilm peptides have a complex mechanism of action, improving our understanding of these mechanisms in biologically relevant situations appears to be important for determining structure–function associations and finally optimizing synthetic antibiofilm peptides for improved antibiofilm potential [120].

### 4.7. Compounds Targeting Metabolism 

Studies into bacterial metabolism show that certain metabolites are necessary for biofilm formation and stability [121]. Recent reports showed small-molecule metabolites and correlated metabolism were essential for biofilm development and dispersal. Pisithkul et al. found that in the early phases of biofilm development, tricarboxylic acid (TCA) cycle activity was increased, iron metabolism and the transport was reorganized, a metabolic shift had occurred from fatty acid biosynthesis to fatty acid degradation, and a switch from acetate to acetoin fermentation took place in *B. subtilis* [122]. In another study by Lu et al., they first evaluated the difference in metabolism between the biofilm and planktonic populations of UTI89 (uropathogenic *E. coli*, UPEC strain) by using mass spectrometry-based targeted and untargeted metabolomic methods, together with cytological imaging, that enabled in identifying the targeted metabolites and related metabolic pathways involved in biofilm development [123]. Interestingly, they could also find distinct changes in both metabolism and phenotypic morphology in two patterns. Moreover, they recognized and categorized 38 differential metabolites and three of the associated metabolic pathways, namely carbohydrate metabolism, amino acid metabolism, and glycerolipid metabolism, were changed typically during biofilm production [123]. In a different study, tea tree oil showed antimicrobial and antibiofilm activity against *S. aureus* (Table 2) and also changed its metabolism by dramatically affecting the expression of genes associated with the pyrimidine metabolism pathway, purine metabolism pathway, glycine, serine, and threonine metabolism pathway, and amino acid biosynthesis pathway [52]. Some research also showed that treatment with exogenous amino acids for example L-arginine was able to control the biofilm formation by repressing the genes that are essential in the formation of *S. mutans* biofilm EPS [124]. Hence, from the recent knowledge about biofilm metabolism, key biofilm metabolites and the chief metabolic pathways could be sorted out. Metabolic engineering into these pathways could be the next essential approach in tackling biofilms. 

### 4.8. EPS Degrading Enzymes for Biofilm Dispersal

Degradation of the EPS by EPS-degrading enzymes such as α-amylase, Dispersin B (DspB), and DNase I is a popular antibiofilm strategy (Table 2). The damage to the basic biofilm component permits more infiltration of antibiotics, thus improving the efficiency of the antibiotic. α-amylase, DspB, and DNase I degrade exopolysaccharides, biofilm EPS, and eDNA, respectively [51,125], which decreases biofilm production as well as degrades mature bacterial biofilms such as *Vibrio cholerae*, *S. aureus*, and *P. aeruginosa* [46]. Polyamine norspermidine and D-amino acids are some of the naturally produced small molecules by bacterial communities that induce the mature biofilm dispersal and also prevent biofilm production in *E. coli* and *S. aureus* [47,48,126]. A different study reported that N-acetylcysteine/NAC and Tween 80 discretely or together with other antibiotics could effectively disperse non-pigmented rapidly growing mycobacteria (RGM) biofilms [49]. Similarly, the researchers in Japan reported that Esp, a serine protease produced by *S. epidermidis* can prevent as well as disperse the *S. aureus* biofilms in vitro. It could likewise repress *S. aureus* nasal colonization in vivo [50]. A similar study by Park et al. also showed that the proteases from *P. aeruginosa* were able to prevent biofilm production and induce dispersal in *S. aureus* [53]. Thus, EPS-degrading enzymes have the potential to be used in biofilm dispersal strategy as an antimicrobial agent.

## 5. Other Therapeutic Approaches

### 5.1. Phage Therapy and CRISPR-Cas 9

Bacteriophage therapy has been used for over 50 years to treat bacterial infections. Recent experimental and clinical studies have demonstrated a remarkable impact on treating both wound biofilm infections as well as device-related infections [127]. The existence of huge numbers of bacteria in the biofilms enables the bacteriophages to rapidly and efficiently infect their host and subsequent multiplication of the bacteriophage. Bacteriophages have many qualities that could make biofilms vulnerable towards them. They are known to produce or induce enzymes that degrade the ECM. They are also able to infect persister cells. Tkhilaishvili et al. have shown that bacteriophages such as Sb-1 enhanced antibiotic activity against biofilm (Table 6) [128]. Moreover, it also degraded the EPS, mainly the polysaccharide content in the biofilms, and targeted the persister cell of *S. aureus* [128]. It is perhaps predictable that bacteriophages can also target the bacteria in biofilms since they prey on bacteria naturally [129]. One of the merits of phage therapy over antibiotic therapy is that the treatment is much more specific. A phage attaches to one specific bacterial strain leaving others unharmed in contrast to the use of antibiotics, which may kill not only harmful bacteria but also helpful bacteria that live in our gut. At the same time, the specificity of phage therapy could also turn into a disadvantage as the matured and naturally produced biofilms could be phage resistant [130].

Clustered regularly interspaced short palindromic repeats and CRISPR-associated protein 9 (CRISPR-Cas9 system) is a novel technology that has been applied for genomic editing in some prokaryotes and eukaryotes [131]. Most recently Zuberi et al. have knockdown the *luxS* gene and fimbriae-associated gene (*fimH*) using the CRISPRi technology for regulating biofilm-associated infections [132,133]. Similarly, Hegde et al. employed the CRISPR/Cas9 system to make an *ompA* (outer membrane protein A) gene knockout in a dominant mosquito microbiota *Cedecea neteri* [134]. The *ompA* mutant displayed a reduction in biofilm formation capability, significantly decreased infection in adults, and was also found to be less prevalent in adults [134]. Thus, CRISPRi seems to be another promising technology for controlling biofilms.

**Table 6:** Some therapeutic approaches effective against biofilms.

Type of Therapy	Target	Biofilm	Type of Assay	References
Bacteriophage therapy	EPS polysaccharide	*S. aureus*	in vitro	[128]
Vaccine (Staphvax)	capsular polysaccharide serotypes (CP5 and CP5)	*S. aureus*	phase III clinical trials	[135]
CRISPR/Cas	*luxS* and *fimH* genes	*E. coli*	in vitro	[132,133]
CRISPR/Cas	ompA	*C. neteri*	in vivo	[134]
Photodynamic therapy	EPS	*S. aureus*	in vitro	[12]
Virus like particles	agr QS system	*S. aureus*	in vivo	[135,136]
Vaccine	pili-S and integration host factor (IHF)	*H. influenzae*	in vivo	[137]
Catalytic antimicrobial robots	kill cells and detach biofilms	*S. mutans*	in vitro	[138]
Nitric oxide-releasing nanoparticles	N/A	*S. aureus*	in vivo	[139]
Arikayce™	inhibition of protein synthesis	*M. avium*	phase III clinical trials	[140]
Fluidsomes™	inhibition of protein synthesis	*P. aeruginosa*	Phase II clinical trials	[140]
Calcium fluoride nanoparticles	various cellular processes	*S. mutans*	in vitro and in vivo	[141]

### 5.2. Vaccines

Antibiotic-resistant species also evolve to colonize and evade host immunity. The approach of targeting biofilms using vaccines poses numerous difficulties in targeting bacterial biofilms since vaccines are specific to a particular microorganism and show large variability in the expression of vaccine-targeted epitopes [4]. Nevertheless, recently several conjugative vaccines have been formulated that target typically conserved EPS components. Staphvax is a conjugative vaccine that comprises polysaccharides and protein that acts on capsular polysaccharide serotypes (CP5 and CP5) in *S. aureus* [135]. The DNABII family of DNA-binding proteins, which consists of the integration host factor (IHF), gives structural integrity to eDNA in biofilms [142]. Studies confirm that antibodies against *E. coli* IHF can bind to DNABII in many bacterial species and destabilize biofilms by releasing individual bacterium. DNA II, when combined with antibiotic therapy, showed a synergistic effect in treating biofilms of numerous bacterial species such as oral bacteria [143], UPEC [144], *P. aeruginosa* [145], and *S. aureus* [146,147] in a murine lung infection model [142]. The next approach is to combine DNABII antibodies with vaccines. A study using IHF and recombinant soluble type IV pili co-administered with an adjuvant in an animal model of otitis media with nontypeable *Haemophilus influenzae* (NTHi) showed early NTHi eradication and prevention of disease [137]. Alternatively, to detect immunogenic mimic of a QS peptide, the usage of Virus-like Particles (VLPs) is increasing. Some results indicate that a diagnosis of VLP-based epitope for the development of a vaccine that targets *agr* signal disruption could be effective against *S. aureus* SSTI [135,136]. Better molecular identification of biofilm-associated genes can advance vaccines development against bacterial infections in the future [135].

### 5.3. Biomaterials and Nanoparticles

Pathogenic biofilms formed on implantable medical devices (IMDs) or human tissues have caused a huge risk in global healthcare. Functionalized biomaterials could be a novel approach to combat and eradicate pre-existing biofilms. Adherence of bacteria is the initial stage of IMD-associated infections that enables bacteria to colonize in the implants. The inhibition of biofilms could be achieved by coating the implants with biomaterials with antifouling and antibacterial properties. Biofilm eradication could be achieved by using nanoparticle (NP)-coated drugs to disperse biofilms [148].

NPs are regarded as a substitute for antibiotics for combating multidrug-resistant and biofilm-associated infections [149]. The biofilm-NP interaction is a three-step process: (1) transport of NPs around the biofilm, (2) attachment of NPs to the biofilm EPS, and (3) penetration of NPs into the EPS and migration within the biofilm through diffusion which might be dependent on the biofilm pore sizes, the charges, hydrophobicity, and the EPS chemical gradient [150]. Drawbacks of antibiotic therapy, such as reduced penetration into the biofilm, could be overcome easily through their nano-formulations that can cross the biological barrier.

Naturally forming and engineered NPs could differ largely in their physicochemical properties including size, shape, and charge [150]. For the last few years, different types of NPs have been used as antibiofilm and antimicrobial metal NPs, organic NPs, green NPs, and their combinations [151]. Several reports exist on NP-based eradication of biofilm communities [152,153,154]. Kulshrestha et al. found the inhibitory effect of CaF2-NPs on genes related to *S. mutans* virulence (*gtfC*, *vicR*, *comDE*, *ftf*, and *spaP*). The study also proposed that CaF2-NPs could also suppress the enzymatic activities related to cell adhesion, glucan synthesis, acid production and tolerance, and quorum sensing that result in biofilm inhibition [141]

NPs such as AuNPs (gold NPs) were also developed in combination with hordenine. Hordenine-AuNPs displayed greater antibiofilm properties on *P. aeruginosa* PAO1, which suggests NPs-delivered natural compounds can be efficiently used in biofilm-related infection [155]. Most recently, Hwang et al., constructed catalytic antimicrobial robots (CARs) that accurately, effectively, and controllably killed, damaged, and detached biofilms [138]. CARs utilizing iron oxide NPs with dual catalytic-magnetic functionality (1) formed free radicals, (2) disrupted biofilm EPS, and (3) eradicated the scrappy biofilm debris using magnetic field-driven robotic assemblies [138]. 

Nitric oxide-releasing nanoparticles (NO NPs) employ various simultaneous antimicrobial mechanisms, so the chances that microbes will develop resistance to NO NPs is low [149]. Schairer et al. compared hydrogel/glass composite NO NP with systemic vancomycin for treating MRSA-infected intramuscular abscesses in a mouse model and found that NO NPs led to a more significant decrease in bacterial survival than vancomycin treated ones [139]. Drug-delivery NPs with targeting ligands have the potential for promoting improved proximity between the individual biofilm cells within the EPS and the nanocarrier. Lipid and polymer NPs are gaining attraction due to their versatility, biocompatibility, targeted/triggered release, and ability to incorporate lipophilic as well as hydrophilic drugs. Many liposomal formulations for biofilm treatment are under development, yet such products are not available on the market. A liposomal formulation containing the antibiotic amikacin, Arikayce™ (Transave, Inc., Monmouth Junction, NJ, USA) is in Phase III clinical trials, Similarly, Fluidsomes™, containing tobramycin, is in a Phase II trial for the treatment of cystic fibrosis-associated respiratory infections. Although there are some examples of antimicrobial catheters, implants, and wound dressings containing AgNPs available for clinical use, antibiofilm strategies are still underdeveloped. Thus, more in vivo studies for therapeutic applications are required regarding the use of nanoparticles [140]. 

### 5.4. Photodynamic Therapy

Photodynamic therapy (PDT) has been utilized for treating several types of viral, fungal, bacterial, protozoan, or even parasitic infections in recent years. It is known that PDT has effectively decreased the clinically important drug-resistant Gram-negative and Gram-positive bacteria [156]. PDT consists of three components: oxygen, visible light, and non-toxic photosensitizers (PS). Since PS-generated ROS can act on several molecules such as DNA, proteins, lipids, biofilm EPS, and even the bacterial cells, PDT appears to have a remarkable possibility in scheming antibiofilm approaches with various targets [157]. Due to its ability of selective binding to the pathogenic cell membranes and particularly targeting the affected tissue for causing extreme harm to microbes while having negligible harm to the host, PDT seems to have some remarkable advantages over conventional methods of treatment [158]. Lately, it was also revealed that PDT could also eliminate the *S. mutans* biofilms-related infections [12]. Li et al. showed that combining photothermal therapy (PTT) and PDT, aided with glutathione oxidation, offered synergistic rapid killing of *S. aureus* biofilm bacteria in vivo than that of PTT or PDT alone [159]. 

## 6. Future Directions

The detection and extraction of newer compounds are now more convenient with the accessibility of innovative techniques [160]. These new compounds can be developed into effective antimicrobial agents for limiting the formation of microbial biofilms on various surfaces. Furthermore, with a better insight into the biofilm formation process, designing novel therapeutic techniques is much easier. The research on the exploration of novel antimicrobial and antibiofilm agents is ongoing. In this article, we summarized the currently available strategies including the antimicrobial agents and the therapeutic approaches. These techniques could provide new hope to move past the antibiotic era. However, there are more possible prospects in the future. We could also combine multiple approaches to find novel strategies to get rid of persistent biofilms, since biofilms are naturally polymicrobial and resilient [161,162]. However, before using each technique it is necessary to understand the merits and demerits of each agent and select the ones that are most efficient in eliminating targeted biofilms by providing limited damage to the host [163]. For many antibiofilm molecules or tools assessed to date, the main reason for their lack of use in clinical practice is due to the gap between the good outcomes achieved in preclinical studies and the evaluation of their clinical potential [164]. Thus, more clinical trials need to be performed for determining the therapeutic application of the antibiofilm agents [163].

Cancer cells and bacterial cells share some similar traits, such as virulence high replication rates, modes of dispersal within the host, and rapid development of drug resistance [9]. Cancer cells and bacteria share several metabolic features and pathways [9]. Some recent studies revealed the relatio164nship between biofilm flora and cancer development [165,166]. It has also been established that the bacteria used host metabolites to form biofilms and to propagate cancer [165]. There has been some success in repurposing some anticancer drugs such as human kinase inhibitors for biofilm inhibition and eradication including the biofilm persisters [167]. Similarly, Mitomycin C, which is an FDA-approved alkylating agent that is being used as a therapeutic agent for treating many cancers, was also found to be effective in killing the persister cells [9]. Thus, repurposing existing drugs not only appears to be an attractive approach in search of innovative anti-biofilm drugs but also saves time and extensive effort of going through de novo drug optimization processes [167]. Subsequently, the anticancer drugs that target metabolism might play a dual role in targeting cancer as well as bacteria. 

Synthetic biology (SB) is a promising interdisciplinary research field that could be used to design and construct newer fabricated devices, artificial metabolic pathways, and organisms, or as well as reform current natural and biological systems, targeting critical problems in health, materials, energy, and the environment [168]. Using several synthetic biological approaches, it is now possible not only to engineer the desired proteins [169] but also to engineer the metabolic pathways [170] or even the biofilm structures [171]. SB also enables us to control biofilms by building quorum-sensing genetic circuits to control biofilms [172,173]. Thus, with better knowledge about biofilms, many novel synthetic biology toolkits could be developed which could expand the potential to control biofilms or even use them for human benefit.

## Figures and Tables

**Table 1:** Classes of antibiofilm agents and their functions.

Antibiofilm Agents	Functions
Class I	penetrate the biofilm EPS and decrease the growth of cells
Class II	interfere with the adherence of bacteria and the formation of biofilm phenotype
Class III	controls both the growth of bacteria with biofilm phenotype as well as the EPS synthesis
Class IV	disperse the mature biofilms

**Table 2:** Different types of antimicrobial agents and their mode of action.

Name of the Compound	Type	Mode of Action	Effective Against	Reference
Triton X-100	surfactant	autolysis, targeting EPS	*S. aureus*	[31,32]
Tween 80	surfactant	N/A	*P. aeruginosa*, *S. aureus*	[30]
Quarternary ammonium compounds	surfactant	Cell lysis and death	several bacteria	[34]
Poloxamer containing non-ionic surfactant	surfactant	EPS metalloproteinase modulation	*P. aeruginosa*	[34]
Rhamnolipids	bio-surfactant	N/A	*S. aureus*, *Salmonella Enteritidis*, and *Listeria monocytogenes*	[36]
EDTA	chelators	damage to cell wall	*P. aeruginosa*	[40]
Chitosan	biomaterial	membrane damage	*P. aeruginosa*	[41]
Secondary metabolite from *Citrus limonoids*	secondary metabolite	quorum sensing	*Vibriyo harveyi*	[42]
Cyclo(l-Tyr-l-Leu)	secondary metabolite	inhibit EPS	*S. epidermidis*	[43]
Cahuitamycins	secondary metabolite	N/A	*A. baumanii*	[44]
Phlorotannin	secondary metabolite	damaging membrane permeability/ cell lysis	MRSA	[45]
α-amylase	enzyme	degrade EPS	MRSA	[46]
Polyamine norspermidine	polyamine	interacts with EPS	*B. subtilis*, *E. coli* and *S. aureus*	[47]
D-amino acids	amino acid	target YqxM	*E. coli*, *S. aureus*	[48]
N-acetylcysteine/NAC	amino acid	degrade EPS polysaccharide	Rapidly growing Mycobacterium	[49]
Esp (Serine protease)	enzymes	degrade EPS protein content	*S. aureus*	[50]
DNase I	enzymes	degrade eDNA	*E. coli*, *S. aureus*	[51]
tea-tree oil	secondary metabolite	metabolism	*S. aureus*	[52]
Protease from *P. aeruginosa*	enzymes	degrade EPS protein content	*S. aureus*	[53]

**Table 3:** Natural compounds as antiquorum sensing molecules in biofilm dispersal.

Compound/Molecule	Mode of Action	Effective Against	Reference
Garlic extracts	inhibits QS	*P. aeruginosa*	[62]
Garlic extracts	inhibit LasR and LuxR	*P. aeruginosa*	[63]
Isolimonic acid	cell-cell signaling	*E. coli*	[64,65]
Isolimonic acid	reduce LuxR DNA binding	*Vibrio* spp.	[65]
Cinnamaldehyde	swimming motility	*E. coli*	[66]
Hordenine	decrease in signaling molecule, inhibition of QS-related genes	*P. aeruginosa*	[67,68]
Autoinducing peptide type I (AIP-I)	inhibit QS	*S. aureus*	[74]
RNAIII-inhibiting peptide (RIP)	inhibit QS	*S. aureus*	[75]
Querentin	decrease LasI/R, RhlI/R expressions	*P. aeruginosa*	[73]

## Data Availability

Not applicable.
